# Structural Characterization and Anti-Ultraviolet Radiation Damage Activity of Polysaccharides from *Helianthus annuus* (Sunflower) Receptacles

**DOI:** 10.3390/molecules30142943

**Published:** 2025-07-11

**Authors:** Xiaochun Chen, Zhiying Wei, Xiaoying Mo, Yantong Lu, Guangjuan Pan, Zhenzhen Pan, Yaohua Li, Hui Tian, Xiaojiao Pan

**Affiliations:** 1 School of Pharmacy, Guangxi University of Chinese Medicine, Nanning 530200, China; 13407724353@163.com (X.C.); weizy@gxtcmu.edu.cn (Z.W.); xy0608ww@163.com (X.M.); 18316062122@163.com (Y.L.); pgjdwyyx@163.com (G.P.); panzz@gxtcmu.edu.cn (Z.P.); liyh2001@gxtcmu.edu.cn (Y.L.); 2 Faculty of Chinese Medicine Science, Guangxi University of Chinese Medicine, Nanning 530222, China; 3 Key Laboratory of TCM Extraction and Purification and Quality Analysis, Guangxi University of Chinese Medicine, Nanning 530200, China; 4 National Demonstration Center for Experimental Chinese Medicine Education, Guangxi University of Chinese Medicine, Nanning 530200, China

**Keywords:** *Helianthus annuus*, receptacle polysaccharides, structural characterization, HaCaT, UV-induced damage

## Abstract

*Helianthus annuus* L. (*H. annuus*) receptacles, a major agricultural by-product generated during seed processing, are currently underutilized. This study aimed to explore the valorization potential of this by-product by extracting *H*. *annuus* receptacles total polysaccharides (HRTP) and characterizing their potential as natural ingredients in ultraviolet (UV)-protective cosmetics. A new purified polysaccharide named *H*. *annuus* receptacles polysaccharide-1 (HRP-1) was isolated, likely exhibiting a backbone of alternating →4)-α-D-GalA-(1→ and →4)-α-D-GalA(6-OCH_3_)-(1→ units, with a weight-average molecular weight (Mw) of 163 kDa. HRTP demonstrated significant protective effects against UV-induced damage in human immortalized keratinocyte (HaCaT) cells by suppressing intracellular reactive oxygen species (ROS) levels and downregulating MAPK-p38/ERK/JNK pathways, thereby inhibiting inflammatory cytokines (IL-1β, IL-6, IL-8, and TNF-α) and matrix metalloproteinases (MMP-1, MMP-3, and MMP-9). Additionally, HRTP exhibited moisturizing properties. These findings highlight *H. annuus* receptacle polysaccharides as sustainable, bioactive ingredients for eco-friendly sunscreen formulations, providing a practical approach to converting agricultural by-products into high-value industrial biomaterials.

## 1. Introduction

The global *Helianthus annuus* L. (*H. annuus*, commonly known as sunflower) industry generates millions of tons of receptacle by-products annually, which are primarily discarded as agricultural residue. While *H. annuus* by-products (e.g., hulls and stalks) have been explored for bioenergy [1,2] and adsorbent applications [3,4], receptacles remain underutilized despite their rich polysaccharide content. Recent studies highlight plant polysaccharides as promising alternatives to synthetic ultraviolet (UV) filters [5,6,7], which face increasing regulatory and environmental concerns due to coral reef toxicity and endocrine disruption risks [8,9,10]. However, most research focuses on edible or medicinal plants, neglecting non-food agricultural by-products—a critical gap in sustainable biomaterial development.

*H. annuus* receptacles contain unique methoxylated pectins [11,12,13,14], but their structural specificity and industrial potential remain unexplored. Previous attempts to valorize these by-products have been limited to crude extracts or food-grade pectin production [15], lacking mechanistic insights into bioactivity. Here, we address these limitations by (1) isolating and structurally characterizing *H. annuus* receptacles polysaccharide-1 (HRP-1) and confirming its predominance in the *H. annuus* total polysaccharides (HRTP). (2) Evaluating HRTP’s UV-protective efficacy through molecular mechanism studies. This work enhances the utilization value of *H. annuus* receptacles by demonstrating their potential as substitutes for other polysaccharides in personal care products, offering UV protection and moisturizing properties.

## 2. Results and Discussion

### 2.1. The Purity Test Results of HRP-1 and the HRP-1 Content in HRTP

HRP-1 was isolated from HRTP using semi-preparative high-performance liquid chromatography (HPLC) equipped with a size-exclusion chromatography column. The purity of HRP-1 and its content in HRTP were subsequently determined by analytical HPLC coupled with an evaporative light scattering detector (ELSD). The purity of HRP-1 was determined to be 99.0% using the peak area normalization technique, which met the requirements of subsequent structural characterization experiments. The HRP-1 content in HRTP was 703.69 ± 11.07 mg/g, which was ~70%, implying that HRP-1 was the main component of HRTP.

The HPLC-ELSD method offers high sensitivity, excellent resolution, and broad versatility, making it particularly suitable for analyzing compounds lacking UV absorption. Since polysaccharides exhibit no significant UV absorption, HPLC-ELSD was employed to quantify the HRP-1 content in HRTP. Methodological investigations revealed a linear relationship between the logarithm of the HRP-1 injection mass (x) and the logarithm of its peak area (y) within a specific concentration range. The linear equation was y = 1.30x + 2.59 (r = 0.993). However, the linear equation did not pass through the origin. As a result, the external standard two-point logarithmic method was applied to determine and calculate the HRP-1 content in HRTP. In quantitative analysis using HPLC-ELSD, it is essential to first establish the relationship between the injection mass of the target analyte and its corresponding peak area [16]. According to the linear equation, we could calculate the injected mass from the measured peak area, thereby determining the content of the analyte in the sample. Thus, validation of this linear relationship constitutes a mandatory step and fundamental prerequisite for quantitative analysis.

### 2.2. Structural Characterization Results of HRP-1

#### 2.2.1. Determination Results of the Molecular Weight of HRP-1

**Figure 1:**
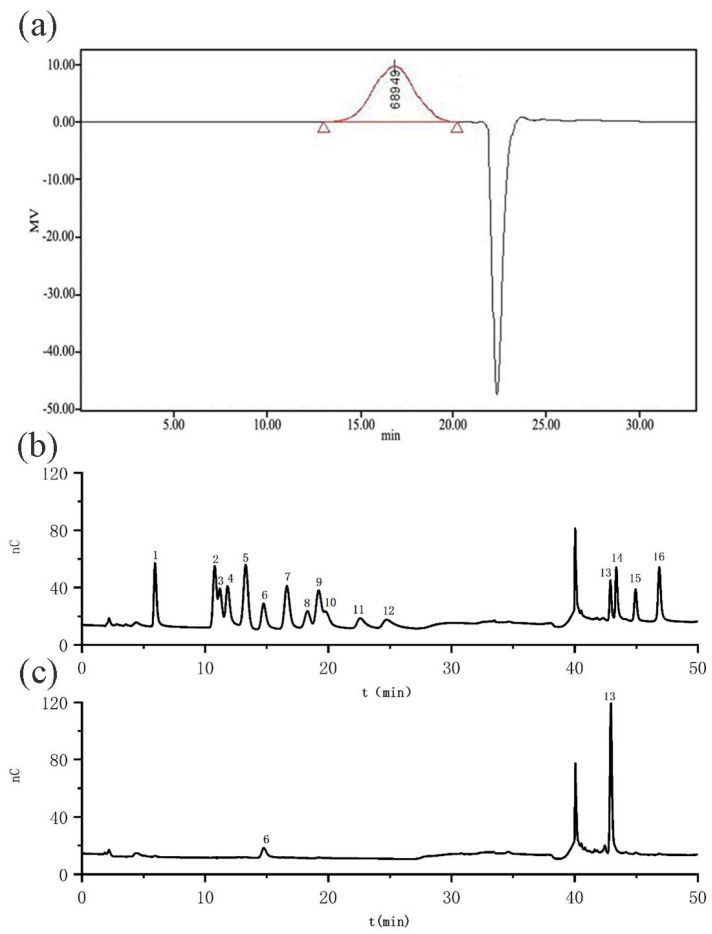
HPLC plot of molecular weight distribution and ion chromatogram of monosaccharide composition of HRP-1. (**a**) HPLC plot of molecular weight distribution; (**b**) Ion chromatogram of standard monosaccharide blends; (**c**) Ion chromatogram of HRP-1. 1. Fuc; 2. GalN; 3. Rha; 4. Ara; 5. GlcN; 6. Gal; 7. Glc; 8. GlcNAc; 9. Xy; 10. Man; 11. Fru; 12. Rib; 13. GalA; 14. GulA; 15. GlcA; 16. ManA. The peaks in (**b**,**c**) at 40 min (RT) are solvent peaks of sodium acetate. The peak in (**a**) at 16.85 min (RT) is HRP-1.

The molecular weight profile of purified HRP-1 was determined by high-performance size-exclusion chromatography (SEC) coupled with a differential refractometer detector (Waters 2410 RID, Waters Corporation, Milford, MA, USA). HRP-1 exhibited a single symmetrical peak at a retention time (RT) of 16.85 min (Figure 1a), indicating homogeneity of the purified polysaccharide. Based on a dextran standard calibration curve: y = −0.5264x + 13.706 (r = 0.996), the weight-average molecular weight (Mw), number-average molecular weight (Mn), and peak molecular weight (Mp) were calculated as 163 kDa, 31 kDa, and 69 kDa, respectively.

#### 2.2.2. Monosaccharide Composition of HRP-1

The monosaccharide composition of HRP-1 was determined using a Thermo Fisher ICS5000 high-pH anion exchange chromatography system (Thermo Fisher Scientific Inc., Waltham, MA, USA). The hydrolyzed polysaccharide (HRP-1 hydrolysate) and monosaccharide standards were analyzed under identical chromatographic conditions (Section 3.4.2).

Comparative analysis of the chromatograms (Figure 1b,c) revealed that galacturonic acid (GalA) was the predominant constituent, eluting at 42.94 min and accounting for 92.2 mol% of the total monosaccharides. This unequivocally classifies HRP-1 as an acidic polysaccharide. A minor peak corresponding to galactose (Gal) was detected at 14.75 min, representing 7.8 mol% of the composition. No other monosaccharides were observed above the detection threshold.

These results indicate that HRP-1 is primarily a galacturonan-type polymer with trace amounts of neutral galactose residues, consistent with pectic polysaccharides commonly found in plant cell walls [17].

#### 2.2.3. Methylation Analysis Resoults of HRP-1

The monosaccharide composition of HRP-1 was dominated by GalA (92.2 mol%), with minor Gal (7.8 mol%). To enable glycosidic linkage analysis via methylation, the carboxyl groups of GalA residues in HRP-1 were reduced to primary alcohols using a catalytic hydrogenation-based method, converting GalA units to Gal derivatives.

GC-MS analysis of the derived partially methylated alditol acetates (PMAAs) revealed five major glycosidic linkages (Table 1): Terminal-Galp (1.8%), →4)-Galp-(1→ (92.8%), →3)-Galp-(1→ (1.4%), →2,4)-Galp-(1→ (1.5%), and →3,6)-Galp-(1→ (2.5%). Critically, due to the absence of isotopic labeling (e.g., deuterium) during reduction, the detected Galp residues cannot be distinguished between (i) native neutral galactose originally present in HRP-1 and (ii) galactose derived from the reduction of galacturonic acid (GalA → Gal).

Based on the predominance of →4)-Galp-(1→ linkages (92.8%) and the original high GalA content, it is inferred that the backbone of HRP-1 primarily consists of →4)-α-GalA-(1→ units, which were converted to →4)-Galp-(1→ linkages after reduction. This structural pattern is characteristic of homogalacturonan (HG)-type pectins, commonly found in plant cell walls [17]. The minor branched linkages (→3)-Galp, →2,4)-Galp, →3,6)-Galp) likely represent side chains or structural variations, potentially contributed by the native galactose residues or modified regions of the galacturonan backbone.

The linear backbone of →4)-α-GalA-(1→ (observed as →4)-Galp post-reduction) may underpin the bioactivity of HRP-1. Previous studies indicate that pectic polysaccharides rich in galacturonan backbones can mitigate UV-induced oxidative stress by scavenging ROS and suppressing pro-inflammatory MAPK pathways [18]. The presence of minor branches may further modulate interactions with cellular receptors or enhance solubility.

In conclusion, methylation analysis provides valuable preliminary insights into the glycosidic linkages of HRP-1, but its limitations, particularly in distinguishing reduced GalA-derived residues from naturally occurring Gal residues, highlight the need for further structural validation using advanced techniques such as NMR spectroscopy. Such analyses will be crucial to establish a definitive correlation between HRP-1’s structural features and its bioactivity. The methylation results are shown in Table 1.

#### 2.2.4. Fourier Transform Infrared (FT-IR) Analysis of HRP-1

**Figure 2:**
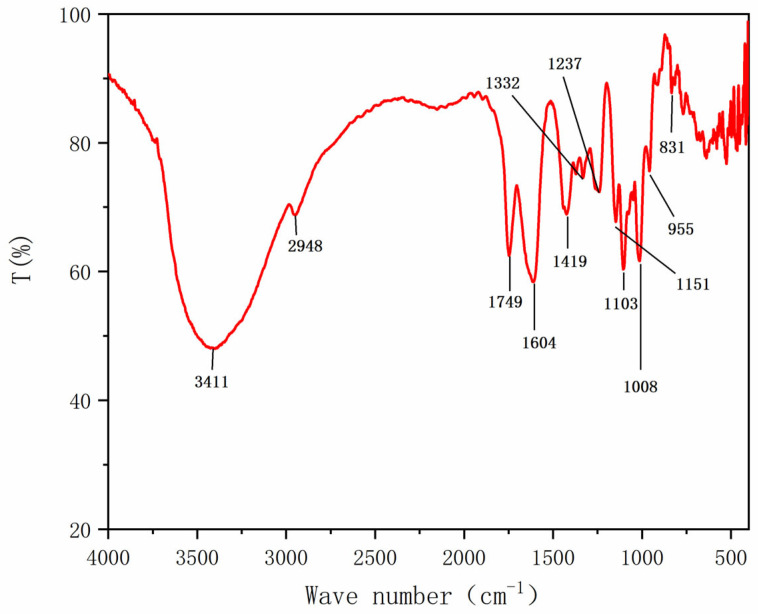
Infrared spectrum of HRP-1.

In the FT-IR spectrum of HRP-1 (Figure 2), the peak at 3411 cm^−1^ was assigned to the ·OH stretching vibration, and that at 2948 cm^−1^ corresponded to the C-H stretching vibration; these were the characteristic peaks of sugar absorption [19]. The peak at 1749 cm^−1^ indicated the presence of ester carbonyl groups (COOR) in HRP-1 [20]. The peak at 1604 cm^−1^ was associated with the asymmetric CO stretch of the carboxylate ion [21], which might be due to water residues. The peak at 1419 cm^−1^ was the symmetrical tensile vibration of COO^−^ [22]. The peak at 1332 cm^−1^ was the deformation vibration of -CH_3_ from galacturonic acid methyl ester [23]. The peak at 1237 cm^−1^ was the C-O stretching vibration. The region between 1000 and 1200 cm^−1^ was considered the fingerprint region. Usually, the asymmetric stretching vibrations of C-O-C and C-O-H were present in this region, which reflected the vibration of the glucopyranose ring [24], and the peaks at 955 cm^−1^ and 831 cm^−1^ corresponded to the α-glycosidic bond [25,26]. The FT-IR results confirmed that HRP-1 exhibited typical polysaccharide structural features, including the presence of uronic acid, methyl uronic acid, and α-linked glycosyl residues. Notable absorption peaks at 3411, 1749, 1419, 1332, and 1237 cm^−1^ were identified as primary recognition peaks for HRP-1.

#### 2.2.5. Nuclear Magnetic Resonance (NMR) Analysis of HRP-1

**Figure 3:**
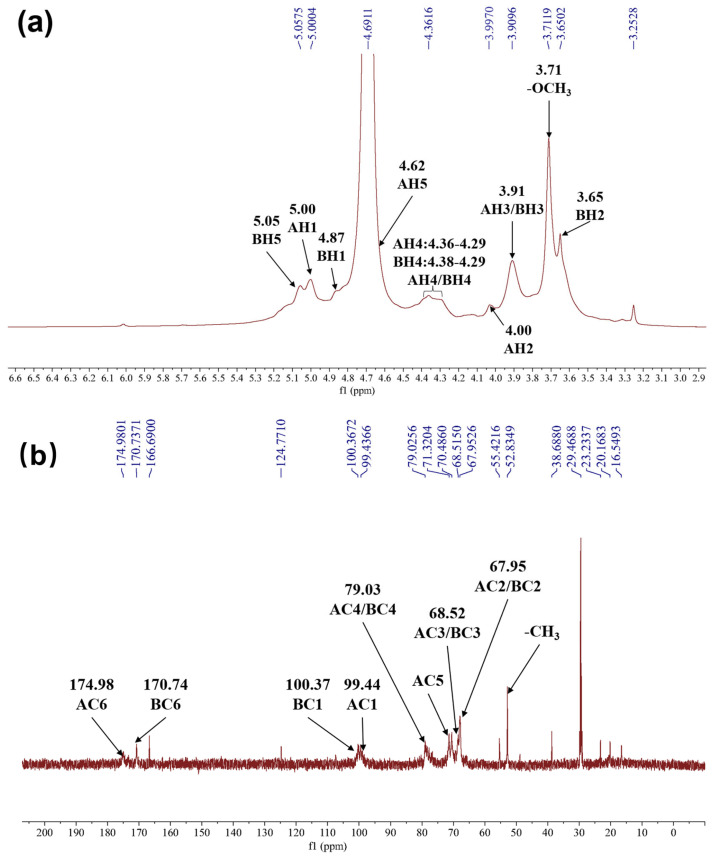
One-dimensional NMR spectra including ^1^H NMR (**a**) and ^13^C NMR (**b**) of HRP-1. AH and AC represent the hydrogen and carbon of the group →4)-α-D-GalA-(1→, respectively. BH and BC represent the hydrogen and carbon of the group →4)-α-D-GalA(6-OCH_3_)-(1→, respectively.

**Figure 4:**
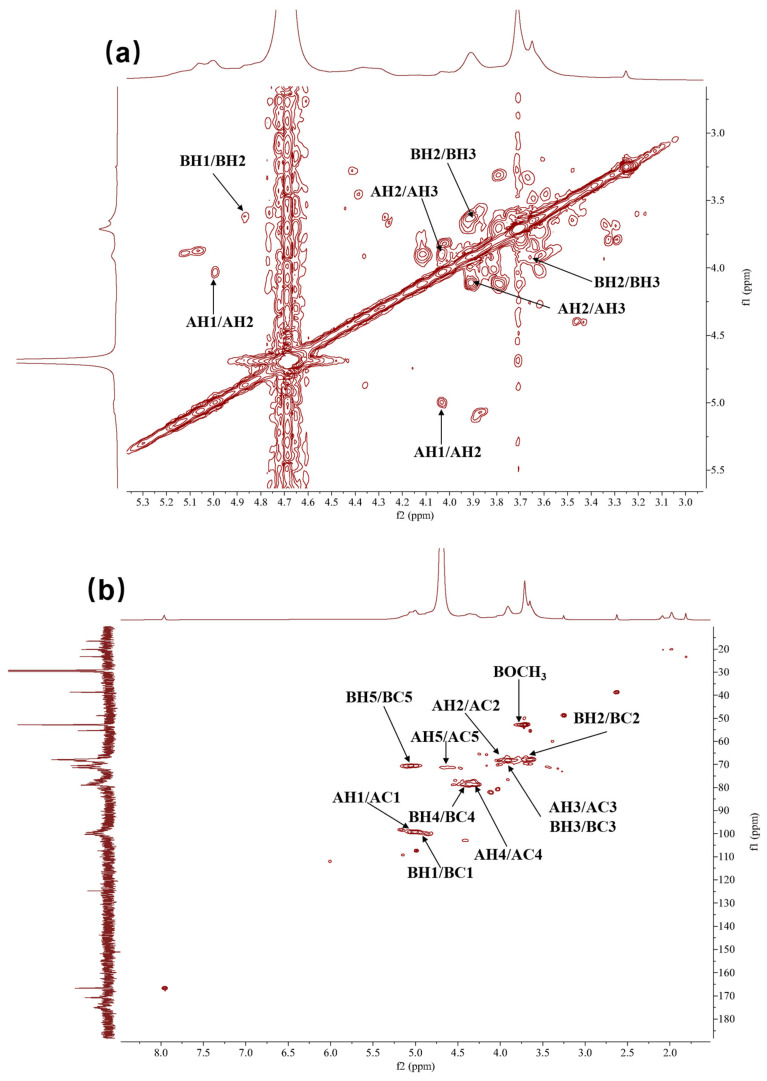

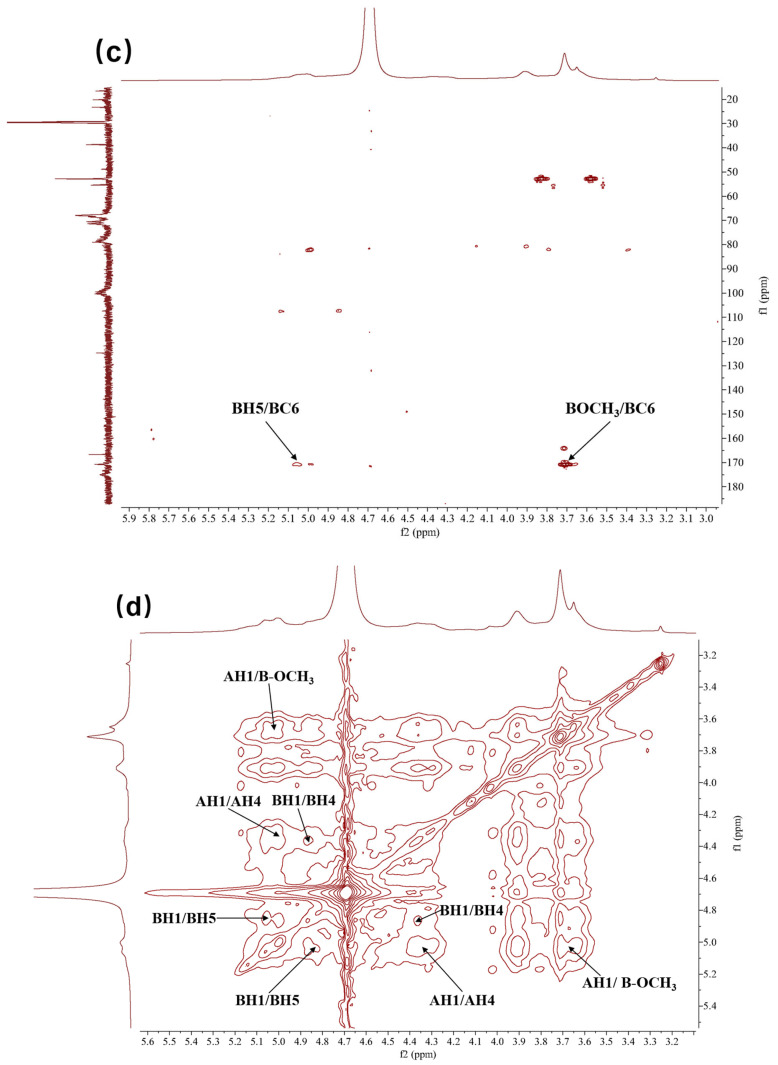
Two-dimensional NMR spectra and structure analysis of HRP-1. (**a**) COSY; (**b**) HSQC; (**c**) HMBC; (**d**) NOESY. AH and AC represent the hydrogen and carbon of the group →4)-α-D-GalA-(1→, respectively. BH and BC represent the hydrogen and carbon of the group →4)-α-D-GalA(6-OCH_3_)-(1→, respectively.

Figure 3 and Figure 4 display HRP-1’s 1D and 2D NMR spectra, respectively, and Table 2 summarizes the carbohydrate signal assignments for HRP-1.

The presence of →4)-α-D-GalA-(1→ confirmed from NMR data and reference [27]. The typical anomeric carbohydrate signals at *δ*_C_ 99.44 (C-1) and *δ*_H_ 5.00 (H-1) ppm were from →4)-D-GalA-(1→ (Figure 3a,b). H-1 (*δ*_H_ 5.00 ppm) and H-2 (*δ*_H_ 4.00 ppm) exhibited δCOSY correlations, confirming the assignment of *δ*_H_ 4.00 ppm to H-2 (Figure 4d). H-2 (*δ*_H_ 4.00 ppm) and H-3 (*δ*_H_ 3.91 ppm) showed COSY correlations, identifying *δ*_H_ 3.91 ppm as the H-3 signal (Figure 4a). The high chemical shift of H-5 (*δ*_H_ 4.62 ppm) indicated that C-6 was a carboxylic acid group, corresponding to the *δ*_C_ 174.98 ppm signal (Figure 3a,b). Furthermore, H-5 (*δ*_H_ 4.62 ppm) was identified based on NOESY correlations with *δ*_H_ 4.00 and 3.91 ppm (Figure 4d). H-1 (*δ*_H_ 5.00 ppm) and H-4 (*δ*_H_ 4.36 ppm) exhibited NOESY correlations, confirming *δ*_H_ 4.36 ppm as the H-4 signal (Figure 4d). The elevated chemical shift of C-4 (*δ*_C_ 79.03 ppm) suggested glycosidic linkage, confirming the structure as →4)-α-D-GalA-(1→ (Figure 3b).

The existence of →4)-α-D-GalA(6-OCH_3_)-(1→ was determined from NMR data and reference [28]. Peaks at *δ*_C_ 100.37 (C-1) and *δ*_H_ 4.87 (H-1) ppm were the typical anomeric carbohydrate signals of →4)-D-GalA(6-OCH_3_)-(1→ (Figure 3a,b); H-l (*δ*_H_ 4.87 ppm) and H-2 (*δ*_H_ 3.65 ppm) exhibited COSY correlations; the *δ*_H_ 3.65 ppm signal corresponds to H-2 (Figure 4a). H-2 (*δ*_H_ 3.65 ppm) and H-3 (*δ*_H_ 3.91 ppm) showed COSY correlations, identifying *δ*_H_ 3.91 ppm as the H-3 signal (Figure 4a). *δ*_H_ 3.71 ppm and *δ*_C_ 52.83 ppm were hydrogen and carbon signals of the -OCH_3_ group. *Δ*_H_ 3.71 ppm and *δ*_C_ 170.74 ppm exhibited HMBC correlations, so that *δ*_H_ 3.71 ppm and *δ*_C_ 52.83 ppm were confirmed to be 6-position carboxylic acid ester signals (Figure 4c). H-5 (*δ*_H_ 5.05 ppm) and C-6 (*δ*_C_ 170.74 ppm) showed HMBC correlations, so it was determined that *δ*_H_ 5.05 ppm was the H-5 signal (Figure 4c). H-1 (*δ*_H_ 4.87 ppm) and H-5 (*δ*_H_ 5.05 ppm) showed NOESY correlations, assigning *δ*_H_ 4.87 ppm to H-1 (Figure 4d). H-1 (*δ*_H_ 4.87 ppm) and H-4 (*δ*_H_ 4.38 ppm) exhibited NOESY correlations, confirming the *δ*_H_ 4.38 ppm as the H-4 signal (Figure 4d). The elevated chemical shift of C-4 (*δ*_C_ 79.03 ppm) indicated glycosidic linkage, confirming the structure as →4)-α-D-GalA(6-OCH_3_)-(1→ (Figure 3b).

In the NMR analysis, the typical carbohydrate signals of →4)-α-D-GalA-(1→ and the hydrogen-carbon signals of →4)-α-D-GalA(6-OCH_3_)-(1→ groups indicated that galacturonic acid methyl ester was also present in HRP-1. The anomeric carbon of →4)-α-D-GalA-(1→ and →4)-α-D-GalA(6-OCH_3_)-(1→ exhibited signals at *δ*_C_ 99.44 and *δ*_C_ 100.37 ppm, respectively, and the height and width of these signals were almost the same (Figure 3b). The equal intensity of the two anomeric carbon signals indicates that their contents are approximately 1:1. This is consistent with an alternating backbone of →4)-α-D-GalA-(1→ and →4)-α-D-GalA(6-OCH_3_)-(1→ units, though sequence regularity requires further validation.

For monosaccharide composition analysis, polysaccharides need to be hydrolyzed, and the hydrolysate of methyl galacturonate is galacturonic acid. Therefore, methyl galacturonate cannot be detected during the monosaccharide composition analysis. In addition, the reduction products of galacturonic acid and methyl galacturonate are both galactose; therefore, the methylation results also cannot reflect the presence of methyl galacturonate in HRP-1. By contrast, because of its high sensitivity and specificity, NMR spectroscopy can directly detect esterification modification, providing more comprehensive information about the structure of polysaccharides. Therefore, NMR analysis is indispensable for characterizing polysaccharide structures.

#### 2.2.6. Characterization of the Primary Structure of HRP-1

**Figure 5:**
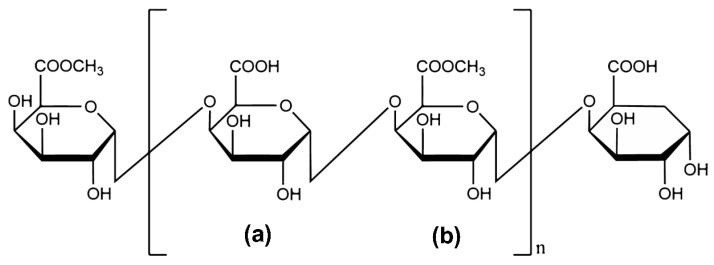
HRP-1 level-1 structure: (**a**) →4)-α-D-GalA-(1→; (**b**) →4)-α-D-GalA(6-OCH_3_)-(1→.

The primary structure of HRP-1 was determined by integrating results from monosaccharide composition, methylation, IR, and NMR analyses, as illustrated in Figure 5. Previous studies have reported the preliminary structural characterization of polysaccharides extracted from *H. annuus* receptacles [29]. However, these studies primarily focused on crude polysaccharides obtained through water extraction and alcohol precipitation, analyzing only via IR, ^1^H NMR, and ^13^C NMR, which confirmed the presence of galacturonic acid and methyl galacturonic acid. In contrast, this work comprehensively analyzed the purified polysaccharide HRP-1, clarifying the primary structure of the main polysaccharide in the *H. annuus* receptacles for the first time.

The likely alternating →4)-α-D-GalA-(1→ and →4)-α-D-GalA(6-OCH3)-(1→ backbone distinguishes HRP-1 from conventional pectins found in citrus or apple, which typically exhibit homogalacturonan domains with random methyl esterification. This structural uniqueness likely enhances HRTP’s hygroscopicity, suggesting superior water-binding capacity for cosmetic formulations. The high methyl ester content (evidenced by FT-IR peaks at 1332 cm^−1^) may further improve skin adhesion, a critical factor for sunscreen efficacy.

Owing to the poor solubility of HRP-1, the quality of its two-dimensional nuclear magnetic resonance (2D NMR) spectra was insufficient to fully corroborate the alternating linkage pattern. In subsequent studies, we plan to optimize NMR characterization through two approaches: (1) Acquiring NMR spectra at elevated temperatures (70–80 °C); (2) Enzymatically digesting HRP-1 with homogalacturonan lyase (HG lyase) to generate oligosaccharide fragments prior to 2D NMR analysis. These strategies will further validate the chemical structure of HRP-1.

#### 2.2.7. Scanning Electron Microscopy (SEM) Analysis Results of HRP-1

**Figure 6:**
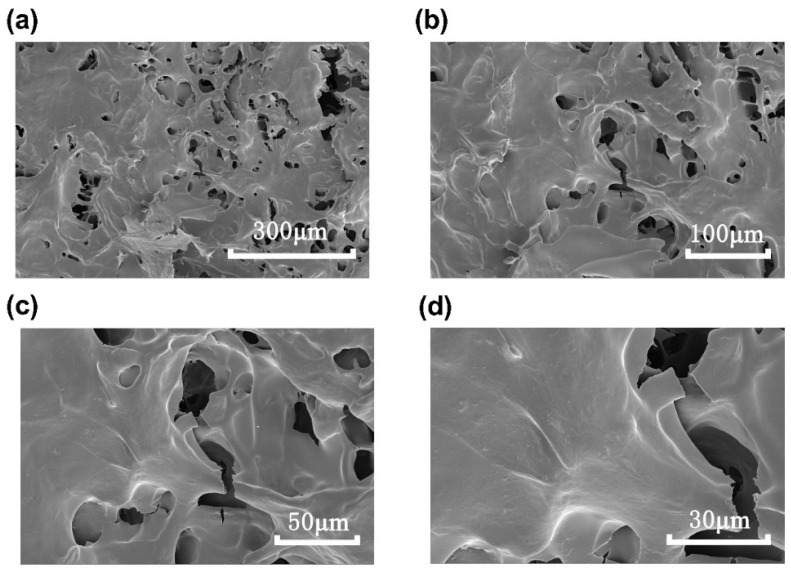
SEM images of HRP-1. (**a**) 500×; (**b**) 1000×; (**c**) 2000×; (**d**) 4000×.

The SEM images of HRP-1 at magnifications of 500×, 1000×, 2000×, and 4000× are shown in Figure 6. The SEM image at 500× (Figure 6a) magnification showed that HRP-1 was lamellar with different thicknesses, and discontinuous pores were present on the surface of the laminates. At 1000× (Figure 6b) and 2000× (Figure 6c) magnifications, the surface of the lamella appeared smooth, with irregular depressions distributed across the surface. At 4000× magnification (Figure 6d), the surface of HRP-1 displayed a smooth texture, but the laminates showed uneven surfaces with prominent cracks.

#### 2.2.8. Atomic Force Microscopy (AFM) Analysis Results of HRP-1

**Figure 7:**
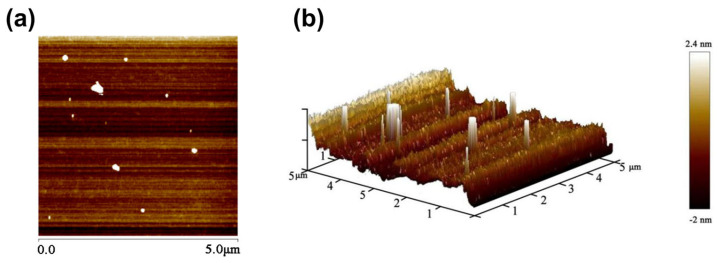
AFM images of HRP-1. (**a**) AFM planar image; (**b**) AFM 3D image.

The microstructural features of the purified polysaccharide HRP-1 were characterized using an atomic force microscope, and the results are shown in Figure 7. The HRP-1 sample was prepared using the drop-casting method and naturally dried on a mica substrate. The AFM planar image revealed that HRP-1 molecules predominantly appeared as irregular white dot-like structures, with no evidence of linear chain-like or branched morphologies (Figure 7a). This suggests that HRP-1 molecules likely underwent partial aggregation during the sample preparation process, rather than existing as individual single chains.

The lateral dimensions of the dot-like structures of HRP-1 were measured using AFM images, ranging from 60 to 300 nm (Figure 7a), with heights ranging from 2.0 to 2.4 nm (Figure 7b). Based on the lateral dimensions and height data, it is evident that the dot-like structures of HRP-1 exhibit an irregular flattened morphology. However, in the AFM 3D image (Figure 7b), these structures appear as elevated stripe-like features. This phenomenon is due to the amplification of the Z-axis scale in the 3D image, which is commonly employed to enhance the visualization of surface topography. The size and morphology of these features suggest that they represent molecular aggregates formed through intermolecular interactions, rather than isolated single-chain molecules. The slow drying process inherent to the drop-casting method likely facilitated the gradual deposition of molecules onto the mica substrate, promoting self-assembly or aggregation via van der Waals forces and hydrogen bonding. Additionally, the ethanol content in the aqueous solution may have further enhanced intermolecular aggregation. Control experiments performed on clean mica surfaces showed no similar dot-like features, confirming that the observed structures were specific to HRP-1.

Overall, the AFM analysis indicates that HRP-1 molecules predominantly exist as aggregates under the drop-casting preparation conditions, rather than as extended single chains. These findings provide valuable insights into the aggregation behavior and intermolecular interactions of HRP-1.

### 2.3. Protective Effect of HRTP Against UV-Induced Damage

#### 2.3.1. Cytotoxicity Resoults

**Figure 8:**
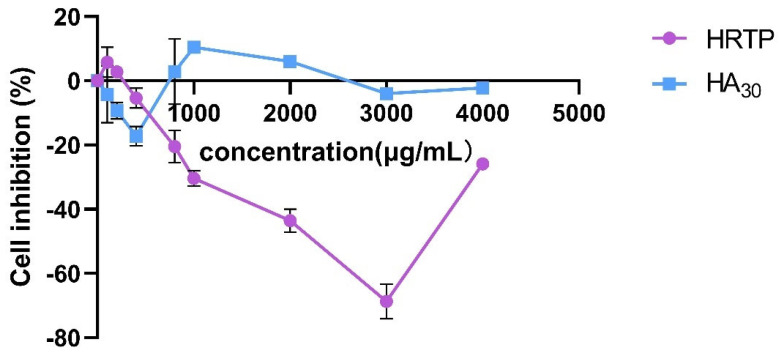
Cytotoxicity of HRTP to HaCaT cells.

The results indicated that HRTP and HA_30_ were non-toxic to HaCaT cells within the concentration range of 0–4000 μg/mL, and IC_50_ could not be determined (Figure 8). Compared to HA_30_, HRTP exhibited a significant proliferative effect on HaCaT cells, with the strongest activity observed at a concentration of 3000 μg/mL, resulting in an inhibition rate of −68.67 ± 5.46%.

Generally, HRTP not only lacked toxic effects on HaCaT cells but also promoted their proliferation at specific concentrations. Polysaccharides have been widely confirmed to promote cell proliferation and wound healing. For instance, algal polysaccharides may enhance wound healing by creating a moist environment that supports cell migration and proliferation [30]. Pine pollen polysaccharides promoted cell proliferation and accelerated wound healing by activating the JAK2-STAT3 signal pathway [31]. The mechanism by which HRTP promotes cell proliferation and whether it can aid in HaCaT wound healing needs to be further studied.

#### 2.3.2. The Preventive and Reparative Effects of HRTP on UV-Induced Damage in HaCaT Cells

**Figure 9:**
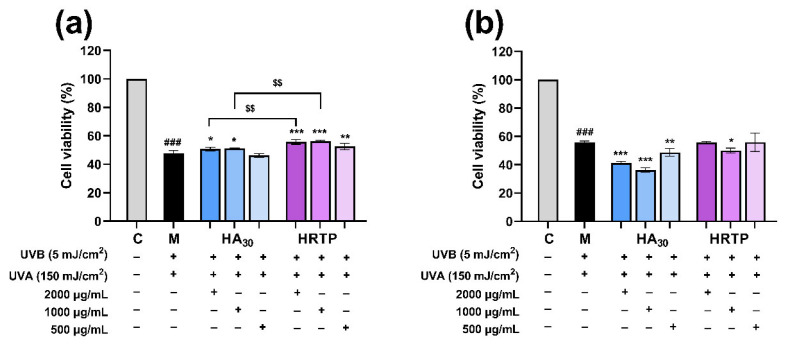
UV-induced damage protection of HRTP on HaCaT cells. (**a**) HRTP’s preventive impact; (**b**) HRTP’s reparative impact. * *p* < 0.05, ** *p* < 0.01, *** *p* < 0.001 as compared to damage model group (M); ^###^ *p* < 0.001 as compared to control group (C); ^$$^ *p* < 0.01, comparison between two designated groups.

In real-life scenarios, HaCaT cells are affected by UVB and UVA radiation, with an energy ratio of approximately 1:30 [32]. Therefore, the HaCaT cell model induced by UVA and UVB radiation was used to investigate the anti-UV damage activity of HRTP. HaCaT cells were irradiated with 150 mJ/cm^2^ UVA and 5 mJ/cm^2^ UVB, respectively, as shown in Figure 9. The survival rate of cells in the model group was ~50% compared to the blank group, validating the success of the skin cell light damage model. Figure 9a demonstrates that the high, medium, and low concentrations of HRTP had a preventive effect against UV-induced damage on HaCaT cells. The survival rates were 55.72 ± 1.69% for high concentration, 56.19 ± 0.64% for medium concentration, and 52.61 ± 2.27% for low concentration, which were all significantly higher than those of the model group (47.92 ± 1.69%, *p* < 0.01). The preventive effect of high and medium concentrations of HRTP was more pronounced than that of HA_30_ at the same concentration (*p* < 0.01). As shown in Figure 9b, compared with the model group, the survival rate of HaCaT cells post-HRTP intervention did not significantly increase, indicating that HRTP exhibited no reparative effect on UV-induced damage.

The experimental results demonstrate that HRTP’s protective effect against UV-induced damage in HaCaT cells is primarily preventive rather than reparative. Numerous studies have shown that polysaccharides possess photoprotective properties, including those derived from bacteria [33] and plants [34,35,36]. Our findings further confirm that polysaccharides have a preventive effect on skin light damage. *H. annuus* receptacles, being abundant agricultural by-products, are inexpensive, readily available, and sustainable. HRTP is characterized by a simple extraction process, high yield, a clear main active ingredient, and low skin toxicity, making it a promising cosmetic raw material.

#### 2.3.3. Levels of Inflammatory Factors, MMPs, and ROS

**Figure 10:**
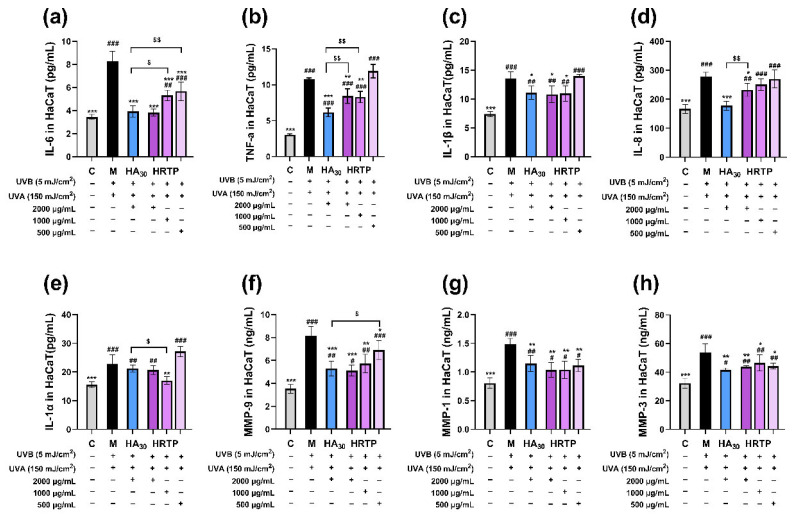
Impact of HRTP on inflammatory factors and MMPs levels in extracellular fluid of HaCaT cells. (**a**) IL-6; (**b**) TNF-α; (**c**) IL-1β; (**d**) IL-8; (**e**) IL-1α; (**f**) MMP-9; (**g**) MMP-1; (**h**) MMP-3. * *p* < 0.05, ** *p* < 0.01, and *** *p* < 0.001, as compared to damage model group (M); ^#^ *p* < 0.05, ^##^ *p* < 0.01, and ^###^ *p* < 0.001, as compared to control group (C); ^$^ *p* < 0.05 and ^$$^ *p* < 0.01, as compared between two designated groups.

After HaCaT cells were irradiated with UVA and UVB, the concentrations of all inflammatory factors and MMPs in the model group’s culture medium were greatly higher than those of the blank group, indicating that UVR induced cell inflammation and increased matrix protease secretion. Figure 10a–e show that HRTP exhibited the strongest inhibitory effect on IL-6 (Figure 10a), with a dose-dependent relationship observed. The inhibitory activities of HRTP and HA_30_ on IL-6 were similar at a concentration of 2000 μg/mL (*p* > 0.05). High concentrations of HRTP (2000 μg/mL) also inhibited TNF-α (Figure 10b), IL-1β (Figure 10c), and IL-8 (Figure 10d); however, its effect was equivalent to or weaker than those of HA_30_ at the same concentration. Figure 10f–h demonstrate that both HRTP and HA_30_ significantly suppressed the UV-induced elevation of MMP-9, MMP-1, and MMP-3 levels (*p* < 0.05 or *p* < 0.01). Among these, HRTP exhibited the strongest inhibitory activity on MMP-9, with a dose-dependent relationship.

**Figure 11:**
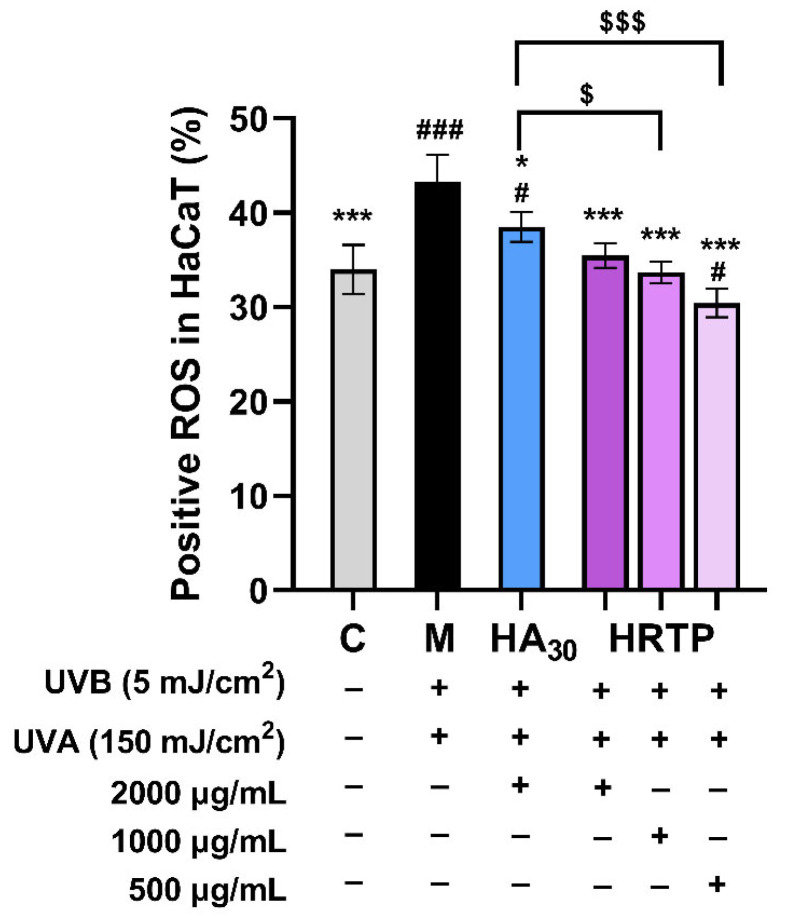
Analysis results of ROS-positive cells in HaCaT cells. * *p* < 0.05 and *** *p* < 0.001, as compared to the damage model group (M); ^#^ *p* < 0.05 and ^###^ *p* < 0.001, as compared to the control group (C); ^$^ *p* < 0.05 and ^$$$^ *p* < 0.001, as compared between two designated groups.

**Figure 12:**
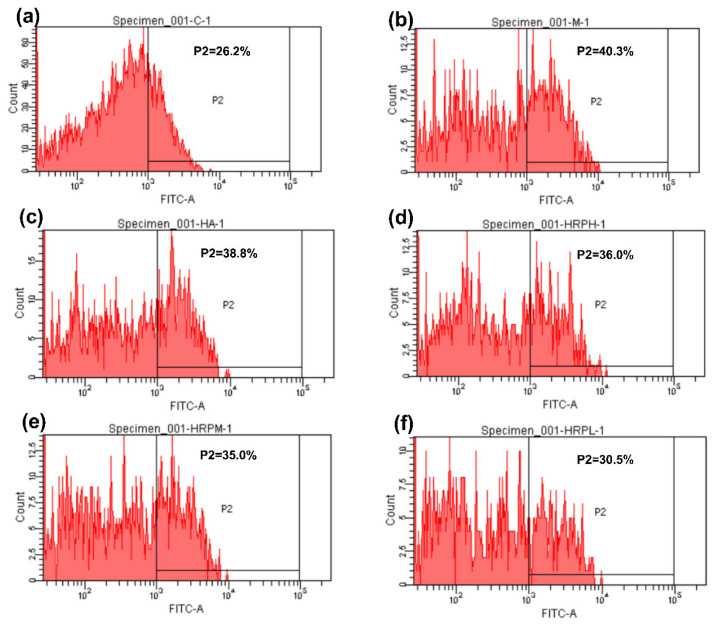
Flow cytometry histograms showing ROS levels in HaCaT cells. (**a**) control group; (**b**) damage model group; (**c**) HA_30_ group; (**d**) 2000 μg/mL HRTP group; (**e**) 1000 μg/mL HRTP group; (**f**) 500 μg/mL HRTP group. Cells with a relative fluorescence intensity higher than 10^3^ are ROS-positive cells, and P2 represents the percentage of ROS-positive cells in the total cells.

Figure 11 and Figure 12 illustrate that UVA and UVB double radiation greatly increased ROS level in HaCaT cells in the model group compared with the blank group (*p* < 0.001). HRTP at concentrations of 500–2000 μg/mL significantly reduced intracellular ROS levels (*p* < 0.001). Notably, the medium (Figure 11 and Figure 12e) and low (Figure 11 and Figure 12f) concentrations of HRTP demonstrated superior ROS scavenging activity compared to HA_30_, with a significantly lower proportion of ROS-positive cells (*p* < 0.05, *p* < 0.001).

In summary, HRTP effectively prevented UV-induced damage in HaCaT cells by significantly reducing inflammatory factors and MMPs, with the most notable inhibitory effects observed on IL-6 and MMP-9. IL-6 is crucial in the inflammatory response, as it recruits and activates inflammatory cells, promotes the release of inflammatory mediators, and plays a role in tissue damage and repair by regulating cell proliferation and differentiation [37]. MMP-9, a complex member of the MMP family, is activated by the MAPK pathway and upregulated by AP-1. Its primary function is to degrade and remodel the extracellular matrix, being mainly secreted by inflammatory cells such as T cells and macrophages [38]. Oxidative stress is a major contributor to UV-induced damage in HaCaT cells, with ROS playing a central role in cell proliferation, apoptosis, and the activation of inflammation-related pathways [39]. HRTP significantly reduced ROS levels in UV-irradiated HaCaT cells, suggesting that this reduction is the primary mechanism underlying its protective effect against UV-induced damage. Furthermore, the reduction in ROS levels likely contributes to the inhibition of inflammatory factors and MMPs.

#### 2.3.4. RT-qPCR Results

**Figure 13:**
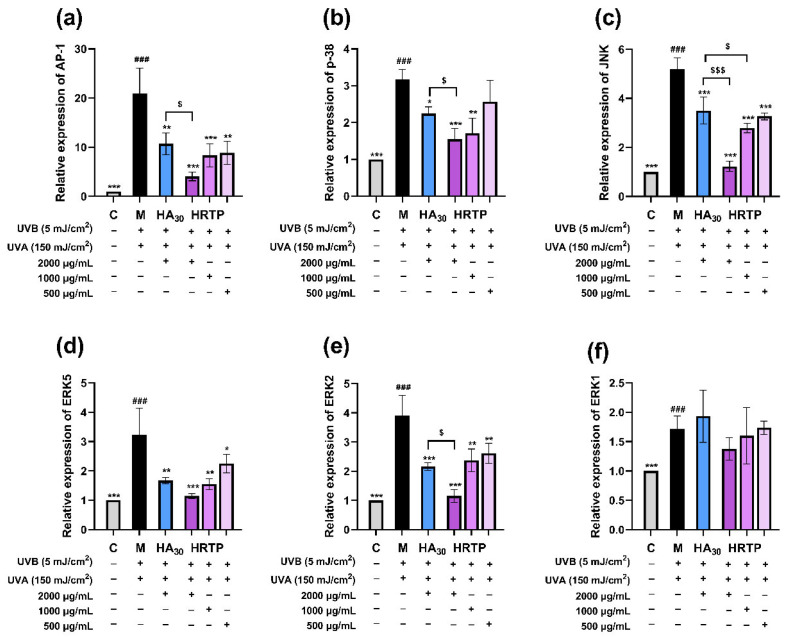
RT-qPCR results of HRTP. (**a**) AP-1 mRNA expression levels; (**b**) p38 mRNA expression levels; (**c**) JNK mRNA expression levels; (**d**) ERK5 mRNA expression levels; (**e**) ERK2 mRNA expression levels; (**f**) ERK1 mRNA expression levels. * *p* < 0.05, ** *p* < 0.01, and *** *p* < 0.001, as compared to damage model group (M); ^###^ *p* < 0.001, as compared to control group (C); ^$^ *p* < 0.05 and ^$$$^ *p* < 0.001, as comapred between two designated groups.

As shown in Figure 13a–e, HRTP at concentrations of 1000–2000 μg/mL significantly reduced the mRNA expression of AP-1, p38, JNK, ERK5, and ERK2 (*p* < 0.01 or *p* < 0.001). The inhibitory activity of HRTP at a high concentration (2000 μg/mL) was more potent than that of HA_30_ for most of the above factors (*p* < 0.05).

In summary, HRTP demonstrated significant protective effects against UV-induced damage in HaCaT cells by suppressing intracellular ROS levels and downregulating MAPK-p38/ERK/JNK pathways, thereby inhibiting inflammatory cytokines (IL-1β, IL-6, IL-8, and TNF-α) and matrix metalloproteinases (MMP-1, MMP-3, and MMP-9).

### 2.4. Hygroscopicity Test Results

**Figure 14:**
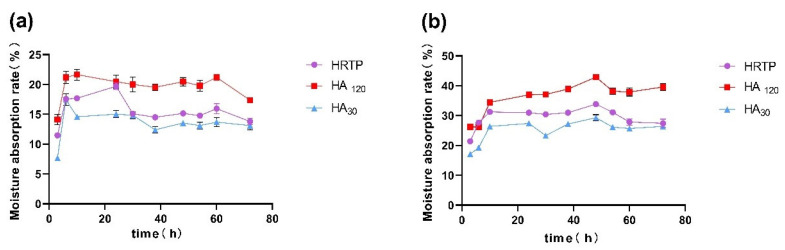
Hygroscopicity of HRTP. (**a**) RH = 42.8%; (**b**) RH = 84.3%.

Figure 14 illustrates that the overall moisture absorption rate of HRTP ranged from 10% to 40%, with its hygroscopic capacity falling between HA_30_ and HA_120_. At a relative humidity (RH) of 42.8% (Figure 14a), HRTP exhibited the strongest moisture absorption activity at 24 h, achieving a moisture absorption rate of 19.70 ± 0.54%. At an RH of 84.3% (Figure 14b), HRTP demonstrated the highest hygroscopicity at 48 h, with a moisture absorption rate of 33.86 ± 0.06%.

The simple ethanol-based purification method (yield: 16.3%, Section 3.2) of HRTP indicates potential for scalable production. Economic analysis indicates that valorizing *H*. *annuus* receptacles could reduce raw material costs by 40–60% compared to polysaccharides derived from marine algae, thereby addressing a critical barrier to the commercialization of green cosmetics. Notably, HRTP’s dual functionality—moisturizing and UV protection—positions it as a versatile cosmetic additive, contrasting with single-function synthetic counterparts.

## 3. Materials and Methods

### 3.1. Materials and Reagents

*H. annuus* receptacles were obtained from Hebei, China; hyaluronic acid, including 300,000 Da (HA_30_) and 1.2 million Da (HA_120_) respectively, were obtained from Ningbo Yinuo Biotechnology Co., Ltd., Ningbo, China; Dextran reference substances with different molecular weights (Dextran T-300, T-150, T-10, T-5 and 180 Da) were purchased from National Institutes for Food and Drug Control, Beijing, China; Standard monosaccharide arabinose (Ara), fructose (Fru), fucose (Fuc), galactose (Gal), galacturonic acid (GalA), glucose (Glc), guluronic acid (GulA), galactosamine hydrochloride (GalN), glucosamine hydrochloride (GlcN), glucuronic acid (GlcA), N-acetyl-D-glucosamine (GlcNAc), mannose (Man), manuronic acid (ManA), rhamnose (Rha), ribose (Rib), xylose (Xyl) and methylation kits were purchased from Jiangsu BoRui Saccharide Biotech Co., Ltd., Yangzhou, China; trifluoroacetic acid (TFA) were obtained from Beijing Yinokai Technology Co., Ltd., Beijing, China; sodium acetate and were obtained from Thermo Fisher Scientific Inc., Waltham, MA, USA; methyl iodide, dimethyl sulfoxide and sodium hydroxide were purchased from Shanghai Adamas Reagent Co., Ltd., Shanghai, China; sodium borohydride and perchloric acid were purchased from Merck Life Science Technology Co., Ltd., Darmstadt, Germany; ethyl acetate was obtained from Wokai Chemical Reagent Co., Ltd., Tianjin, China; acetic anhydride was obtained from Shanghai Chemical Reagent Co., Ltd., Shanghai, China; potassium bromide was obtained from National Pharmaceutical Group Chemical Reagent Co., Ltd., Shanghai, China. In addition, human immortalized keratinocyte (HaCaT) cells were procured from Hunan Fenghui Biotechnology Co., Ltd., Changsha, China; fetal bovine serum (FBS) was obtained from Procell Life Science & Technology Co., Ltd., Wuhan, China; dulbecco’s modified eagle medium (DMEM) was provided by Gibco, Waltham, MA, USA; dimethyl sulfoxide (DMSO), phosphate buffered saline (PBS), 0.25% trypsin, penicillin–streptomycin and Cell Counting Kit-8 (CCK-8) was obtained from Beijing Solarbio Science & Technology Co., Ltd., Beijing, China; kit for determination of tumor necrosis factor-α (TNF-α), interleukin-1α (IL-1α), interleukin-1β (IL-1β), interleukin-6 (IL-6), interleukin-8 (IL-8), and matrix metalloproteinases (MMPs), including matrix metalloproteinase-1 (MMP-1), matrix metalloproteinase-3 (MMP-3) and matrix metalloproteinase-9 (MMP-9) were all procured from Quanzhou Ruixin Biotechnology Co., Ltd., Quanzhou, China The Reactive Oxygen Species (ROS) Assay Kit was procured from Beyotime Biotechnology; Monzol^TM^ Reagent Pro, MonScript^TM^ RTIII Allin-One Mix with dsDNase, and MonAmp^TM^ ChemoHS Specificity Plus qPCR Mix (None ROX) were procured from Monad Biotech Co., Ltd., Suzhou, China.

### 3.2. Preparation of HRTP and HRP-1

First, the *H. annuus* receptacles (~25 cm in diameter) with the seeds removed were dried at 50 °C and crushed into a powder. Second, 100.00 g of powder was heated and extracted twice with 20 times the amount of water for 1 h each time. The extract was filtered through medical gauze using a vacuum pump. The filtrate was then collected and combined. Concentration under reduced pressure and freeze-drying were performed on the filtrate to obtain 42.41 g of *H. annuus* receptacle water extract. The extraction rate was 42.41%. Next, 10.00 g of water extract was dissolved in 100 mL of pure water and the alcohol content was adjusted to 60% by adding ethanol. Let it stand at room temperature for 2 h, then the solution was centrifuged at 2795× *g* (r = 10 cm) for 10 min, and the precipitate was collected and redissolved with an appropriate amount of water. Ethanol was then added to adjust the alcohol content to 60%. Next, alcohol precipitation was performed twice. The polysaccharide precipitates were collected, washed twice with ethanol, and freeze-dried to obtain 3.84 g of light brown flake-like powder of HRTP with a yield of 16.28%. A suitable amount of HRTP powder was dissolved in pure water and purified by semi-preparative liquid chromatography (LC; LC-20AR chromatograph, Shimadzu Corporation, Kyoto, Japan). Chromatographic conditions were as follows: chromatographic column = Thermo Scientific SEC-300 (Thermo Fisher Scientific Inc., Waltham, MA, USA) (300 mm × 7.8 mm, 5 μm); mobile phase = 100% pure water; flow rate = 2 mL/min; detection wavelength = 210 nm; column temperature = 25 °C; injection volume = 400 μL. The samples were repeatedly injected, and the fractions were collected according to the shape of the absorption peak and RT. The samples were concentrated under reduced pressure and freeze-dried to obtain the purified polysaccharide in milky white powder form, named HRP-1.

### 3.3. Purity Test and Determination of the HRP-1 Content in HRTP

#### 3.3.1. Purity Test of HRP-1

The purity of HRP-1 was tested using a 1260 Infinity high-performance liquid chromatograph with the Alltech 3300 evaporative light scattering detector (ELSD; Agilent Technologies Inc., Santa Clara, CA, USA). Chromatographic conditions were as follows: chromatographic column = Thermo Scientific SEC-300 (7.8 mm × 300 mm, Thermo Fisher Scientific Inc., Waltham, MA, USA); mobile phase = 80% pure water + 20% acetonitrile; column temperature = 25 °C; flow rate = 0.8 mL/min; ELSD air flow rate = 1.5 L/min; drift tube temperature = 60 °C; gain value = 4.

#### 3.3.2. Determination of HRP-1 in HRTP

HRP-1 was precisely weighed, and a standard solution of 2.00 mg/mL concentration was prepared using distilled water. The HRTP (~0.05 g) was precisely weighed and transferred into a conical flask. Subsequently, 10 mL of distilled water was introduced into the flask, ultrasonically treated for 40 min, cooled, and centrifuged. Next, the supernatant was precisely diluted twice and used as the sample solution. Under the chromatographic conditions reported in Section 3.3.1, 5 and 10 μL of the HRP-1 reference solution and 5 μL of the HRTP sample solution were injected, respectively. According to the external standard logarithmic two-point method, HRP-1 reference solutions with different injection masses were used, and *a* and *b* in the linear equation were calculated. The HRP-1 content in the HRTP was then calculated. The logarithmic linear equation is as follows:

lg(A) = *a* × lg (V × C) + *b*

where A is the peak area, V is the injection volume, and C is the injection concentration.

### 3.4. Structural Characterization of HRP-1

#### 3.4.1. Determination of the Molecular Weight of HRP-1

The relative molecular weight of HRP-1 was determined using 2695 high-performance liquid chromatography with a 2410 differential refractometer (Waters Corporation, Milford, MA, USA). Dextran control samples with different molecular weights and HRP-1 were dissolved in the mobile phase to form a sample solution of an appropriate concentration. The chromatographic conditions were as follows: The column used was an Ultrahydrogel™ Linear (300 mm × 7.8 mm). The mobile phase consisted of 0.1 M sodium nitrate, with a flow rate set at 0.5 mL/min and a column temperature maintained at 40 °C. The standard curve was plotted using the RT of the glucan peak as the abscissa and the lgMw as the ordinate. The Mw, Mn, and Mp of HRP-1 were calculated using a standard curve and polysaccharide molecular weight analysis software [40].

#### 3.4.2. Monosaccharide Composition Analysis of HRP-1

The monosaccharide composition analysis of HRP-1 was performed according to the reference with slight modifications [41]. The monosaccharides present in HRP-1 were determined using high-pH anion exchange chromatography due to their electrochemical activity and ionization in strong alkaline solutions.

Preparation of the standard solution: 16 types of monosaccharide reference substances (Ara, Fuc, Fru, GalN, GlcN, Gal, Glc, GlcNAc, GalA, GulA, GlcA, Man, ManA, Rib, Rha, and Xyl) were precisely weighed and dissolved in water to prepare a mixed reference solution.

Preparation of the sample solution: 5 mg of samples were accurately weighed in ampoules, and 2 mL of 3 M trifluoroacetic acid (TFA) was added to hydrolyze the sample for 3 h at 120 °C. The solution from acid hydrolysis was transferred to a tube and evaporated under nitrogen. Subsequently, 5 mL of deionized water was added, and the mixture was vortexed thoroughly to prepare the sample solution. A 50 μL aliquot of the sample solution was added to 950 μL of deionized water and then centrifuged. The supernatant was collected for IC analysis.

Chromatographic conditions: instrument = ICS5000 high pH anion exchange chromatography with an electrochemical detector (Thermo Fisher Scientific Inc., Waltham, MA, USA); chromatographic column = Dionex Carbopac^TM^ PA20 (3 mm × 150 mm); mobile phases = H_2_O (A), 15 mM NaOH (B), and 15 mM NaOH + 100 mM NaAc (C); gradient elution [0–18 min, A/B/C = 98.8:1.2:0; 18.1–30 min, A/B/C = 50:50:0; 30.1–46 min, A/B/C = 0:0:100; 46.1–50 min, A/B/C = 0:100:0]; injection volume = 25 μL; flow rate = 0.3 mL/min; column temperature = 30 °C.

Results calculation: Monosaccharide amounts were determined using absolute quantification, and molar ratios were calculated based on their molar masses.

#### 3.4.3. Methylation Analysis of HRP-1

**Figure 15:**
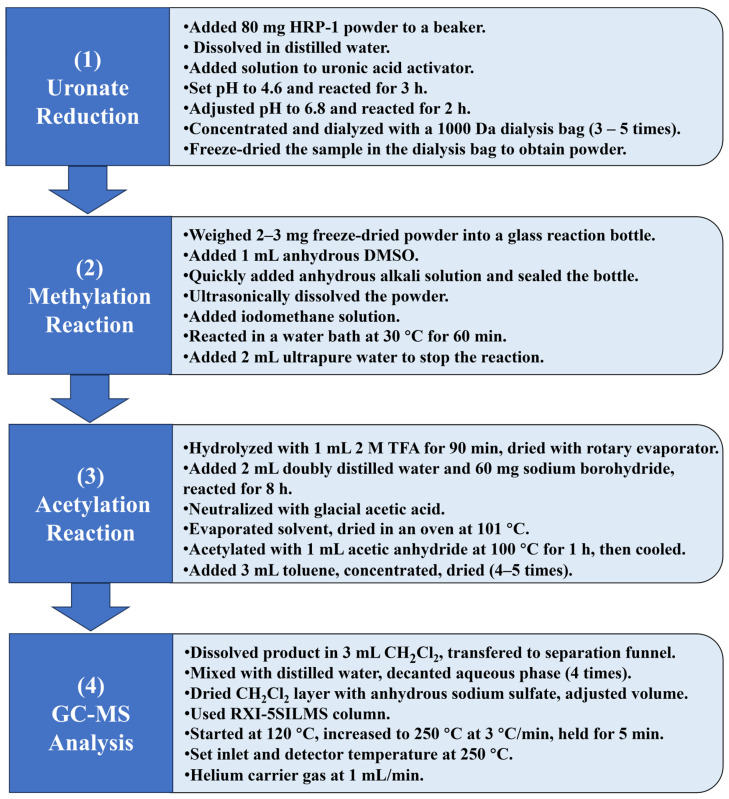
Flow chart of methylation analysis assay.

The methylation analysis experimental process is as shown in Figure 15 [42]. The sample’s reduction experiment was performed using a uronic acid reduction instrument (BR-HYY-001, BoRui Saccharide Biotech Co., Ltd., Yangzhou, China). The acetylation product samples were analyzed via gas chromatography-mass spectrometry (GC-MS; 6890-5973 gas chromatography-mass spectrometer, Agilent Technologies Inc., Santa Clara, CA, USA).

#### 3.4.4. FT-IR Analysis of HRP-1

FT-IR spectroscopy was conducted using a Thermoelectric iS10 infrared spectrometer (Thermo Fisher Scientific Inc., Waltham, MA, USA). After the HRP-1 and potassium bromide particles were dried, they were mixed in a 1:20 ratio, ground using a mortar, and pressed into a transparent sheet for detection. The FT-IR spectrum was recorded across a wavenumber range of 4000 to 400 cm^−1^, utilizing a resolution of 4 cm^−1^. The spectrum was cumulatively scanned 30 times, with water and CO_2_ interference removed during scanning [43].

#### 3.4.5. NMR Analysis of HRP-1

The structure of HRP-1 was analyzed using a 600 MHz Avance III NMR spectrometer (Bruker Scientific Technology Co., Ltd., Billerica, MA, USA). A 20 mg sample of HRP-1 was dissolved in 0.55 mL of 99.9% D_2_O and transferred to a 5 mm NMR tube. One-dimensional (1D) NMR (i.e., ^1^H and ^13^C spectra) and two-dimensional (2D) NMR [i.e., homonuclear ^1^H/^1^H correlated (COSY) spectrum, homonuclear ^1^H/^1^H enhanced (NOESY) spectrum, heteronuclear ^1^H/^13^C single quantum correlated (HSQC) spectrum, and heteronuclear ^1^H/^13^C multibond correlated (HMBC) spectrum] of the sample were analyzed. The structure of HRP-1 was elucidated.

#### 3.4.6. SEM Analysis of HRP-1

A Nova NanoSEM450 ultrahigh-resolution scanning electron microscope (FEI Company, Hillsboro, OR, USA) was used for analysis. Approximately 5 mg of HRP-1 was immobilized onto a conductive carbon film with a double-sided adhesive and allowed to dry. The film was then inserted into the sample chamber of the ion-sputtering instrument, and gold was sprayed onto it for approximately 40 s. After the sample was taken out, the surface morphology of HRP-1 was observed using a scanning electron microscope at an accelerated voltage of 5 kV [44].

#### 3.4.7. AFM Analysis of HRP-1

An appropriate amount of HRP-1 was dissolved in an aqueous ethanol solution (20 µg/mL) and then subjected to agitation in a water bath at 60 °C for 120 min. Subsequently, the treated solution (10 μL) was placed on a mica sheet and dried at 25 °C for 12 h. AFM was then conducted in the tapping mode at room temperature using a Dimension ICON atomic force microscope (Bruker Scientific Technology Co., Ltd., USA). The scanning range was 5 × 5 µm [45].

### 3.5. Study on the Protective Effect of HRTP Against UV-Induced Damage

#### 3.5.1. Cell Culture

HaCaT cells were cultured in DMEM supplemented with 10% FBS and 1% penicillin-streptomycin at 37 °C and 5% CO_2_. Upon cell density reaching 80–90% confluency, the cells were dissociated using 0.25% trypsin and then passed in a 1:5 ratio.

#### 3.5.2. Cytotoxicity

HaCaT cells in the logarithmic growth phase were inoculated at a density of 2 × 10^4^ cells/well in 96-well plates for 12 h. The HRTP group and positive control HA_30_ group were prepared using phosphate-buffered saline (PBS) to obtain a sample stock solution of 5 mg/mL and diluted with a culture medium. The final concentrations of each sample administered to the cells were 0, 100, 200, 400, 800, 1000, 2000, 3000, and 4000 μg/mL, respectively. Subsequent to incubation with the sample for 24 h, absorbance was assessed at 450 nm employing the CCK-8 technique. The inhibition rate was computed according to the following formula:

Cell inhibition (%) = [1 − (absorbance of the sample − absorbance of blank control)/(absorbance of cell control − absorbance of blank control)] × 100%

#### 3.5.3. Ultraviolet Radiation (UVR) Procedure

(1) UVR for Assessing the Preventive Effect

HaCaT cells in the logarithmic growth phase were inoculated at a density of 2 × 10^4^ cells/well in 96-well plates for 12 h. The blank model, HA_30_ positive control, and HRTP groups were established. The mother liquid dissolved in PBS was diluted into cells at appropriate concentrations, and the final concentrations of each sample were 2000, 1000, and 500 μg/mL, respectively. Then, the cells were exposed to the samples for 1 h. Following this, the lids of the 96-well plates were removed, and the cells were exposed to UVA and UVB radiation at doses of 150 and 5 mJ/cm^2^, respectively. Post-irradiation, the cells were rinsed once with PBS and then incubated in the medium for 24 h. Subsequently, the absorbance was measured at 450 nm by the CCK-8 assay, and the survival rate was calculated. The radiation dose, measured in mJ/cm^2^, was calculated by multiplying the radiation intensity (mw/cm^2^) by the exposure time (seconds). The cell viability was calculated using the following equation [46]:

Cell viability (%) = [(absorbance sample − absorbance of blank control)/(absorbance of cell control − absorbance of blank control)] × 100%

(2) UVR for Assessing the Reparative Effect

HaCaT cells were inoculated in a 96-well plate at a density of 2 × 10^4^ cells/well for 12 h. Then, the cells were covered with 100 μL of PBS for radiation. The experimental groups and radiation methods were the same as those used to assess the preventive effects. Following irradiation, the PBS was aspirated, and the sample stock solution was diluted with the medium to achieve the desired concentrations, with final concentrations of 2000, 1000, and 500 μg/mL, respectively. The cells were incubated in the sample solutions for 24 h, followed by absorbance measurement at 450 nm using the CCK-8 assay, and cell viability was calculated [46].

#### 3.5.4. ELISA

HaCaT cells were seeded into 6-well plates at a density of 6 × 10^5^ cells/well and cultured for 12 h. The UVR procedure for cells was the same as that used to assess the preventive effect in Section 3.5.3. The final concentration of HA_30_ was set at 2000 μg/mL, while HRTP was prepared at concentrations of 2000, 1000, and 500 μg/mL. Following treatment, the cell culture medium was collected, and the concentrations of IL-1α, IL-1β, IL-6, IL-8, TNF-α, MMP-9, MMP-3, and MMP-1 were measured according to the manufacturer’s instructions.

#### 3.5.5. Determination of ROS Levels

ROS levels were assessed using the same cell cultivation, sample handling, and UV radiation procedures described in the ELISA protocol. Post-treatment, cells were incubated with the fluorescent probe 2′,7′-dichlorodihydrofluorescein to assess ROS levels. The specific procedures, instruments, and analysis software used were identical to those described in reference [46].

#### 3.5.6. Quantitative Real-Time PCR (RT-qPCR)

HaCaT cells were inoculated at a density of 1.2 × 10^6^ cells/dish in a 6-cm petri dish for 12 h. The cell concentration and UVR procedure were the same as those mentioned in Section 3.5.3. RNA extraction and reverse transcription were performed on the collected cells. Primers were designed based on gene sequences retrieved from the National Center for Biotechnology Information (NCBI) database using Primer Premier v5.0. Glyceraldehyde-3-phosphate dehydrogenase (GAPDH) was used as the reference gene. RT-qPCR was used to detect the mRNA expression of activating protein-1 (AP-1), c-Jun N-terminal kinase (JNK), p38 protein kinase (p38), and extracellular regulated protein kinase (ERK). The specific procedures, instruments, and analysis software used were identical to those described in reference [46]. The RT-qPCR primer sequences utilized in this study are presented in Table 3.

### 3.6. Study of Hygroscopicity

The hygroscopicity test was conducted according to reference [47], with modifications to the relative humidity (RH) conditions and sampling intervals. A number of clean weighing bottles were weighed using a balance after being marked with numbers. The mass of each weighing bottle was recorded as M_0_. Appropriate amounts of HA_30_, HA_120_, and HRTP (0.03–0.05 g) were weighed and added to the weighing bottles, respectively. The weight of the weighing bottle and sample was noted as M_1_. The bottles were immediately covered after weighing and then placed in a dryer with RH of 42.8% and 84.3%, respectively. The caps of the weighing bottles were opened, and the bottles were weighed after placing them in a dryer for 3, 6, 10, 24, 30, 36, 48, 54, 60, and 72 h, respectively. The total mass (m_x_) of the weighing bottle and sample at each time was recorded. Each group consisted of three replicates. The moisture absorption rates of samples were calculated as follows:

Moisture absorption rate (%) = (m_x_ − m_1_)/(m_1_ − m_0_) × 100%

### 3.7. Statistical Analysis

Each experiment comprised a minimum of three biological replicates and three technical replicates. All variables are presented as mean ± SD and analyzed by SPSS version 28 software. A univariate analysis of variance (ANOVA) was employed to evaluate statistical differences between groups, with significance set at *p* < 0.05.

## 4. Conclusions

This study demonstrates the potential of polysaccharides derived from *H. annuus* receptacles as sustainable and functional ingredients for UV-protective cosmetics. Using a simplified ethanol-based purification process, HRTP was obtained with a yield of 16.3%. Its predominant component, HRP-1, accounted for approximately 70% of the total polysaccharide content and was identified as a novel pectic polysaccharide featuring a likely alternating backbone of →4)-α-D-GalA-(1→ and →4)-α-D-GalA(6-OCH_3_)-(1→ units. HRTP exhibited significant bioactivity, including ROS scavenging, suppression of inflammatory cytokines, and inhibition of matrix metalloproteinases, which collectively contribute to its preventive efficacy against UV-induced skin damage. Moreover, HRTP demonstrated intermediate hygroscopic properties between HA_30_ and HA_120_, achieving a moisture absorption rate of 33.9% at 84.3% relative humidity, making it suitable as a natural moisturizing agent.

This work underscores the value of *H. annuus* receptacles as an abundant, low-cost biomass resource, promoting their circular utilization and reducing reliance on synthetic and marine-derived materials. HRTP’s simple extraction process, cost-effectiveness, and multifunctional properties position it as a promising candidate for eco-friendly cosmetic formulations. Furthermore, the study aligns with green chemistry principles by offering a scalable approach to agricultural by-product valorization, contributing to sustainability and environmental protection. Future research should focus on optimizing HRTP formulations for topical applications and assessing their environmental impact during large-scale production.

## Figures and Tables

**Table 1:** HRP-1 methylated sugar alcohol acetyl ester (PMAA) results analysis.

RT (min)	Methylated Sugar	Mass Fragments (*m*/*z*)	Molar Ratio (%)	Type of Linkage
17.520	2,3,4,6-Me_4_-Galp	45, 71, 87, 101, 117, 129, 145, 161, 205	1.8	Galp-(1→
20.780	2,3,6-Me_3_-Galp	45, 87, 99, 101, 113, 117, 129, 131, 161, 173, 233	92.8	→4)-Galp-(1→
21.381	2,4,6-Me_3_-Galp	45, 87, 99, 101, 117, 129, 161, 173, 233	1.4	→3)-Galp-(1→
24.849	3,6-Me_2_Galp	45, 87, 99, 113, 129, 173, 189, 233	1.5	→2,4)-Galp-(1→
27.988	2,4-Me_2_-Galp	45, 87, 117, 129, 159, 189, 233	2.5	→3,6)-Galp-(1→

**Table 2:** H NMR and ^13^C NMR data of HRP-1 in D_2_O.

Position	*δ*_C_, Type	*δ*_H_	^1^H-^1^H COSY	HMBC	NOESY
→4)-α-D-GalA-(1→					
1	99.44, CH	5.00	4.00		4.36, 3.91, 4.00
2	67.95, CH	4.00	5.00, 3.91		
3	68.52, CH	3.91	4.00		
4	79.03, CH	4.36–4.29			
5	71.32, CH	4.62			4.00, 3.91
6	174.98, C				
→4)-α-D-GalA(6-OCH_3_)-(1→					
1	100.37, CH	4.87	3.65		3.65, 3.91, 4.38, 5.05
2	67.95, CH	3.65	4.87, 3.91		
3	68.52, CH	3.91	3.65		
4	79.03, CH	4.38–4.29			
5	70.49, CH	5.05		170.74	
6	170.74, C				
OCH_3_	52.83, OCH_3_	3.71		170.74	5.05, 4.87, 4.38, 3.91

**Table 3:** Primer sequences for RT-qPCR analysis.

Gene	Direction	Primer Pair Sequence (5′ → 3′)
GAPDH	F	AATCAAGTGGGGCGATGCTG
	R	GCAAATGAGCCCCAGCCTTC
AP-1	F	TCTGGGAAGTGAGTTCGCCT
	R	ATGCCTCCCGCACTCTTACT
p38	F	GATTTTGGACTGGCTCGGCA
	R	CAGTCAACAGCTCGGCCATT
JNK	F	CCAGTCAGGCAAGGGATTTGTTAT
	R	TCTTTGGTGGTGGAGCTTCTG
ERK5	F	AGCTATCTAAGTCACAGGTGGAGG
	R	CAAAGCCAACACCGTAGCCA
ERK2	F	CGCCGAAGCACCATTCAAGT
	R	CAGCGCCTCCCTTGCTAGA
ERK1	F	CAACACCACCTGCGACCTTA
	R	TGGACTTGGTATAGCCCTTGGAG

## Data Availability

Data are contained within the article.

## References

[1] Gude V.G., Martinez-Guerra E. (2018). Green Chemistry with Process Intensification for Sustainable Biodiesel Production. Environ. Chem. Lett..

[2] Ebrahimian E., Denayer J.F.M., Aghbashlo M., Tabatabaei M., Karimi K. (2022). Biomethane and Biodiesel Production from Sunflower Crop: A Biorefinery Perspective. Renew Energy.

[3] Anastopoulos I., Ighalo J.O., Adaobi Igwegbe C., Giannakoudakis D.A., Triantafyllidis K.S., Pashalidis I., Kalderis D. (2021). Sunflower-Biomass Derived Adsorbents for Toxic/Heavy Metals Removal from (Waste) Water. J. Mol. Liq..

[4] Nazemi S., Mohmmadkhani S., Yeganeh J. (2016). Sunflower Wasted Biomass as a Remarkable Adsorbent for Removal of Heavy Metals from Waste Waters. Int. J. Health Stud..

[5] Li L., Huang T., Lan C., Ding H., Yan C., Dou Y. (2019). Protective Effect of Polysaccharide from *Sophora japonica* L. Flower Buds against UVB Radiation in a Human Keratinocyte Cell Line (HaCaT Cells). J. Photochem. Photobiol. B.

[6] Ye Y., Ji D., You L., Zhou L., Zhao Z., Brennan C. (2018). Structural Properties and Protective Effect of *Sargassum fusiforme* Polysaccharides against Ultraviolet B Radiation in Hairless Kun Ming Mice. J. Funct. Foods.

[7] Li H., Li Z., Peng L., Jiang N., Liu Q., Zhang E., Liang B., Li R., Zhu H. (2017). *Lycium barbarum* Polysaccharide Protects Human Keratinocytes against UVB-Induced Photo-Damage. Free Radic. Res..

[8] Conway A.J., Gonsior M., Clark C., Heyes A., Mitchelmore C.L. (2021). Acute Toxicity of the UV Filter Oxybenzone to the Coral *Galaxea fascicularis*. Sci. Total Environ..

[9] Miller I.B., Pawlowski S., Kellermann M.Y., Petersen-Thiery M., Moeller M., Nietzer S., Schupp P.J. (2021). Toxic Effects of UV Filters from Sunscreens on Coral Reefs Revisited: Regulatory Aspects for “Reef Safe” Products. Environ. Sci. Eur..

[10] Carstensen L., Beil S., Börnick H., Stolte S. (2022). Structure-Related Endocrine-Disrupting Potential of Environmental Transformation Products of Benzophenone-Type UV Filters: A Review. J Hazard. Mater..

[11] Muñoz-Almagro N., Garrido-Galand S., Taladrid D., Moreno-Arribas M.V., Villamiel M., Montilla A. (2022). Use of Natural Low-methoxyl Pectin from Sunflower By-products for the Formulation of Low-sucrose Strawberry Jams. J. Sci. Food Agric..

[12] Peng X., Yang G., Shi Y., Zhou Y., Zhang M., Li S. (2020). Box–Behnken Design Based Statistical Modeling for the Extraction and Physicochemical Properties of Pectin from Sunflower Heads and the Comparison with Commercial Low-Methoxyl Pectin. Sci. Rep..

[13] Tan J., Hua X., Liu J., Wang M., Liu Y., Yang R., Cao Y. (2020). Extraction of Sunflower Head Pectin with Superfine Grinding Pretreatment. Food Chem..

[14] Muthusamy S., Manickam L.P., Murugesan V., Muthukumaran C., Pugazhendhi A. (2019). Pectin Extraction from *Helianthus annuus* (Sunflower) Heads Using RSM and ANN Modelling by a Genetic Algorithm Approach. Int. J. Biol. Macromol..

[15] Qiao Z., Han L., Liu X., Dai H., Liu C., Yan M., Li W., Han W., Li X., Huang S. (2021). Extraction, Radical Scavenging Activities, and Chemical Composition Identification of Flavonoids from Sunflower (*Helianthus annuus* L.) Receptacles. Molecules.

[16] Chen M., Liu C., Shen Y., Zou J., Zhang Z., Wan Y., Yang L., Jiang S., Qian D., Duan J. (2021). A Powerful HPLC-ELSD Method for Simultaneous Determination of Fecal Bile Acids in T2DM Rats Interfered by Sanhuang Xiexin Tang. J. Chromatogr. Sci.

[17] Frosi I., Balduzzi A., Moretto G., Colombo R., Papetti A. (2023). Towards Valorization of Food-Waste-Derived Pectin: Recent Advances on Their Characterization and Application. Molecules.

[18] Deng R., Wang F., Wang L., Xiong L., Shen X., Song H. (2023). Advances in Plant Polysaccharides as Antiaging Agents: Effects and Signaling Mechanisms. J. Agric. Food Chem..

[19] Hu J., Yao W., Chang S., You L., Zhao M., Chi-Keung Cheung P., Hileuskaya K. (2022). Structural Characterization and Anti-Photoaging Activity of a Polysaccharide from *Sargassum fusiforme*. Food Res. Int..

[20] Chen W., Zhu X., Ma J., Zhang M., Wu H. (2019). Structural Elucidation of a Novel Pectin-Polysaccharide from the Petal of *Saussurea laniceps* and the Mechanism of Its Anti-HBV Activity. Carbohydr. Polym..

[21] Pappas C.S., Malovikova A., Hromadkova Z., Tarantilis P.A., Ebringerova A., Polissiou M.G. (2004). Determination of the Degree of Esterification of Pectinates with Decyl and Benzyl Ester Groups by Diffuse Reflectance Infrared Fourier Transform Spectroscopy (DRIFTS) and Curve-Fitting Deconvolution Method. Carbohydr. Polym..

[22] Chen J., Zhang X., Huo D., Cao C., Li Y., Liang Y., Li B., Li L. (2019). Preliminary Characterization, Antioxidant and α-Glucosidase Inhibitory Activities of Polysaccharides from Mallotus Furetianus. Carbohydr. Polym..

[23] Chetouani A., Follain N., Marais S., Rihouey C., Elkolli M., Bounekhel M., Benachour D., Le Cerf D. (2017). Physicochemical Properties and Biological Activities of Novel Blend Films Using Oxidized Pectin/Chitosan. Int. J. Biol. Macromol..

[24] Zhao X., Li J., Liu Y., Wu D., Cai P., Pan Y. (2017). Structural Characterization and Immunomodulatory Activity of a Water Soluble Polysaccharide Isolated from *Botrychium ternatum*. Carbohydr. Polym..

[25] Xie F., Zhang H., Nie C., Zhao T., Xia Y., Ai L. (2021). Structural Characteristics of Tamarind Seed Polysaccharides Treated by High-Pressure Homogenization and Their Effects on Physicochemical Properties of Corn Starch. Carbohydr. Polym..

[26] Li F., Feng K.-L., Yang J.-C., He Y.-S., Guo H., Wang S.-P., Gan R.-Y., Wu D.-T. (2021). Polysaccharides from Dandelion (*Taraxacum mongolicum*) Leaves: Insights into Innovative Drying Techniques on Their Structural Characteristics and Biological Activities. Int. J. Biol. Macromol..

[27] Zhao Z., Dai H., Wu X., Chang H., Gao X., Liu M., Tu P. (2007). Characterization of a Pectic Polysaccharide from the Fruit of *Ziziphus jujuba*. Chem. Nat. Compd..

[28] Li J., Ai L., Yang Q., Liu Y., Shan L. (2013). Isolation and Structural Characterization of a Polysaccharide from Fruits of *Zizyphus jujuba* Cv. Junzao. Int. J. Biol. Macromol..

[29] Ezzati S., Ayaseh A., Ghanbarzadeh B., Heshmati M.K. (2020). Pectin from Sunflower By-Product: Optimization of Ultrasound-Assisted Extraction, Characterization, and Functional Analysis. Int. J. Biol. Macromol..

[30] Kumar M., Kumar D., Garg Y., Mahmood S., Chopra S., Bhatia A. (2023). Marine-Derived Polysaccharides and Their Therapeutic Potential in Wound Healing Application—A Review. Int. J. Biol. Macromol..

[31] Wang C., Shang H., Cui W., Zhou F., Zhang S., Wang X., Gao P., Wei K., Zhu R. (2022). Pine Pollen Polysaccharides Promote Cell Proliferation and Accelerate Wound Healing by Activating the JAK2-STAT3 Signaling Pathway. Int. J. Biol. Macromol..

[32] Fourtanier A., Moyala D., Seite S. (2012). UVA Filters in Sun-Protection Products: Regulatory and Biological Aspects. Photochem. Photobiol. Sci..

[33] Guerreiro B.M., Freitas F., Lima J.C., Silva J.C., Reis M.A.M. (2021). Photoprotective Effect of the Fucose-Containing Polysaccharide FucoPol. Carbohydr. Polym..

[34] Li Q., Wang D., Bai D., Cai C., Li J., Yan C., Zhang S., Wu Z., Hao J., Yu G. (2020). Photoprotective Effect of *Astragalus membranaceus* Polysaccharide on UVA-Induced Damage in HaCaT Cells. PLoS ONE.

[35] Long Y., Wang W., Zhang Y., Du F., Zhang S., Li Z., Deng J., Li J. (2023). Photoprotective Effects of *Dendrobium nobile* Lindl. Polysaccharides against UVB-Induced Oxidative Stress and Apoptosis in HaCaT Cells. Int. J. Mol. Sci..

[36] Guo L., Yang Y., Pu Y., Mao S., Nie Y., Liu Y., Jiang X. (2024). Dendrobium Officinale Kimura & Migo Polysaccharide and Its Multilayer Emulsion Protect Skin Photoaging. J. Ethnopharmacol.

[37] Hirano T. (2021). IL-6 in Inflammation, Autoimmunity and Cancer. Int. Immunol..

[38] de Moura J.P., de Moura Fernandes E.P., Lustoza Rodrigues T.C.M., Messias Monteiro A.F., de Sousa N.F., Dos Santos A.M.F., Scotti M.T., Scotti L. (2023). Targets Involved in Skin Aging and Photoaging and their Possible Inhibitors: A Mini-review. Curr. Drug Targets.

[39] Fang M., Lee H.-M., Oh S., Zheng S., Bellere A.D., Kim M., Choi J., Kim M., Yu D., Yi T.-H. (2022). *Rosa davurica* Inhibits Skin Photoaging via Regulating MAPK/AP-1, NF-ΚB, and Nrf2/HO-1 Signaling in UVB-Irradiated HaCaTs. Photochem. Photobiol. Sci..

[40] Zhang X., Wang L., Xie F., Yaseen A., Chen B., Zhang G.L., Wang M.K., Shen X.F., Li F. (2021). A Polysaccharide TKP-2–1 from *Tamarindus indica* L.: Purification, Structural Characterization and Immunomodulating Activity. J. Funct. Foods.

[41] Niu L., Wu Y., Liu H., Wang Q., Li M., Jia Q. (2021). The Structural Characterization of a Novel Water-Soluble Polysaccharide from Edible Mushroom *Leucopaxillus giganteus* and Its Antitumor Activity on H22 Tumor-Bearing Mice. Chem. Biodivers..

[42] Li H., Liu M., Liu Z., Cheng L., Li M., Li C. (2023). Purification, Structural Characterization, and Antitumor Activity of a Polysaccharide from Perilla Seeds. Int. J. Mol. Sci..

[43] Zhang H., Zou P., Zhao H., Qiu J., Regenstein J.M., Yang X. (2021). Isolation, Purification, Structure and Antioxidant Activity of Polysaccharide from Pinecones of *Pinus koraiensis*. Carbohydr. Polym..

[44] Hu Z., Zhao K., Chen X., Zhou M., Chen Y., Ye X., Zhou F., Ding Z., Zhu B. (2023). A Berberine-Loaded *Bletilla striata* Polysaccharide Hydrogel as a New Medical Dressing for Diabetic Wound Healing. Int. J. Mol. Sci..

[45] Huang X., Li T., Li S. (2023). Encapsulation of Vitexin-Rhamnoside Based on Zein/Pectin Nanoparticles Improved Its Stability and Bioavailability. Curr. Res. Food Sci..

[46] Mo X., Chen X., Pan X., Lu Y., Pan G., Xie J., Pan Z., Li L., Tian H., Li Y. (2024). Protective Effect of *Helianthus Annuus* Seed Byproduct Extract on Ultraviolet Radiation-induced Injury in Skin Cells. Photochem Photobiol.

[47] Shi C., Fang Y., Liu Z., Wang Y., Shen L., Zhao L. (2025). Effect of Moisture Sorption and Lactose Type on Tablet Quality: A Hygroscopicity Study between Lactose Powder and Tablets. Mol. Pharm..

